# Gastrointestinal changes in paediatric malnutrition that may impact on nutrition choice

**DOI:** 10.3389/fped.2025.1523613

**Published:** 2025-03-10

**Authors:** Rosan Meyer, Lauren Arpe, Aydan Kansu, Veronica Kelly, Keith Lindley, Mairéad O'Meara, Maria del Carmen Rivero, Suzanne van Zundert, Saioa Vicente-Santamaría, Orjena Žaja, Elena Oliveros, Leanne Olivier, Koen Joosten

**Affiliations:** ^1^ Department of Medicine, KU Leuven, Leuven, Belgium; ^2^ Gastroenterology Department, Great Ormond Street Hospital, London, United Kingdom; ^3^ Department of Pediatric Gastroenterology, Hepatology & Nutrition, Ankara University School of Medicine, Ankara, Türkiye; ^4^ Department of Pediatrics, Children’s Health Ireland, Dublin, Ireland; ^5^ Department of Pediatric Gastroenterology and Nutrition, Hospital Virgen de la Macarena, Seville, Spain; ^6^ Department of Nutrition and Dietetics, Amsterdam University Medical Centre, Emma Children’s Hospital, Amsterdam, Netherlands; ^7^ Department of Pediatrics, Hospital Universitario Ramón y Cajal, Madrid, Spain; ^8^ Sestre Milosrdnice University Hospital Center, University of Zagreb, Zagreb, Croatia; ^9^ Nutrition Science, Abbott Nutrition, Granada, Spain; ^10^ Medical Affairs & Research, Nutrition International, Abbott, Maidenhead, United Kingdom; ^11^ Department of Intensive Care Neonatology & Pediatrics, Erasmus MC-Sophia Children’s Hospital, Rotterdam, Netherlands

**Keywords:** disease-related malnutrition, gastrointestinal function, children, nutritional support, malabsorption, diarrhoea, peptide-based enteral therapy

## Abstract

Undernutrition is defined as “a condition resulting from imbalanced nutrition or abnormal utilization of nutrients.” In this paper, the term malnutrition is used to refer to undernutrition. Malnutrition may be driven by poor socioeconomic conditions or by disease, and it is estimated that disease-related malnutrition (DRM) impacts up to 28% of hospitalized children in Europe. Malnutrition results in alterations in gastrointestinal function that lead to malabsorption of macro- and micro-nutrients. It can lead to altered gut motility and a deficiency of stomach acid, which can result in intestinal colonization by pathogens, causing diarrhoea and high burdens of intestinal infection. The presence of compromised gastrointestinal function in children with DRM is critical as it negatively impacts the efficacy of nutritional support and recovery. When choosing novel strategies and nutritional therapies for malnourished children, consideration should be given to gut-protective interventions that promote better treatment tolerance. When breastmilk is unavailable, whole protein feeds are currently considered as first-line treatment for malnutrition in children with a normal functioning gastrointestinal tract. However, peptide-based feeds have been associated with improved gastrointestinal tolerance and absorption, reduced diarrhoea, reduced inflammation, improved growth and have restored gut integrity compared with free amino acid and whole-protein feeds. At a recent meeting, experts in this area have identified significant research gaps in the literature on peptide-based feeds in children and possible gaps in clinical practice. Whilst the group acknowledges that further work is needed, this paper provides an overview on this topic to further drive research in this area.

## Introduction

Paediatric undernutrition is defined by the European Society for Paediatric Gastroenterology, Hepatology & Nutrition (ESPGHAN) as “a condition resulting from imbalanced nutrition or abnormal utilization of nutrients which causes clinically meaningful adverse effects on tissue function and/or body size/composition with subsequent impact on health outcomes” (1). Whilst the term malnutrition encompasses both undernutrition and overnutrition, malnutrition is extensively used to refer to undernutrition and will be used in this context throughout. The World Health Organization (WHO) has established anthropometric indicators (z scores) for malnutrition diagnosis. Undernutrition presents in four broad forms: wasting (low weight-for-height), often indicating recent or severe weight loss due to inadequate food intake and/or infectious disease; stunting (low height-for-age), linked to chronic or recurrent undernutrition and factors like poverty, poor maternal health and nutrition, frequent illness and/or inappropriate early-life care; and underweight (low weight-for-age), which may also involve stunting, wasting, or both; there is another form of malnutrition that refers to deficiencies in vitamins and minerals, called hidden hunger (2). In 2022, 149 million children under the age of 5 years were estimated to be stunted and 45 million were estimated to be wasted (2).

Disease-related malnutrition (DRM) impacts up to 28% of hospitalized children in Europe, depending on the country, patient population and diagnostic criteria used (3, 4). In addition, DRM has been shown to be associated with increased healthcare costs for hospitalized children (5). Malnutrition can affect gastrointestinal function, resulting in alterations in intestinal blood flow, pancreatic exocrine insufficiency, villus atrophy and increased intestinal permeability leading to loss of digestive enzymes, malabsorption of carbohydrate, fat, protein and other nutrients, secondary lactose intolerance, loss of absorption in the colon and diarrhoea (6–9). As a result of this, the impaired absorption of available nutrients during the treatment of malnutrition together with the risk of refeeding-mediated diarrhoea can affect the speed and extent of recovery (10). Furthermore, malnutrition can lead to altered gut motility and a deficiency of stomach acid which, combined with impaired cell immunity, can result in intestinal colonization by pathogens causing diarrhoea and high burdens of intestinal infection (6, 7, 11, 12).

Novel strategies and therapies are needed to provide nutritional support for affected individuals, to improve the effect of nutritional intervention and also to target other aspects of malnutrition such as inflammation and malabsorption (10, 12). A UK national survey across four tertiary paediatric centres involving 191 children with a median age of 19 months reported that 17% were on amino acid-based feeds and 83% were on extensively hydrolysed feeds for conditions other than cow's milk allergy. Whilst specialized feeds are commonly used in clinical practice, the evidence-based research supporting the use of these different types of feed was scarce (13).

This article focuses on key gastrointestinal changes that occur in malnutrition, the impact that some of these changes can have when providing nutritional support to malnourished paediatric patients, including the challenge of tolerance, and nutritional considerations when making feed choices (when breastmilk is insufficient or not available) in children over 1 year of age, utilizing a methodological literature search to review the available evidence and extract relevant published articles.

## Search methodology

The PubMed, EMBASE and Dimensions databases were searched up to August 2023 using the search terms: peptide-based/hydrolysed/semi-elemental formula/feed AND malnutrition AND children/pediatric/paediatric; peptide-based/hydrolysed/semi-elemental formula/feed AND undernutrition AND children/pediatric/paediatric; peptide-based/hydrolysed/semi-elemental formula/feed AND failure to thrive/faltering growth AND children/pediatric/paediatric; peptide-based/hydrolysed/semi-elemental formula/feed AND malabsorption AND children/pediatric/paediatric.

Initially, 660 articles were identified in the PubMed and EMBASE databases and 500 articles in the Dimensions database. These were reviewed for relevance by two reviewers, with a third to resolve any disputes. Inclusion criteria included: articles in English language, full text articles, randomized controlled trials, case cohort studies, retrospective observational studies and review papers. Exclusion criteria included: studies in infants, adolescent or adults, preclinical studies, no peptide-based feeds studies or studies evaluating irrelevant outcomes for the purpose of this paper. After considering the inclusion and exclusion criteria, 643 and 498 articles were excluded and the number of relevant articles to 19, comprising 12 clinical studies and 7 review articles. This included all publications that conformed to the inclusion criteria. Supplementary Figure S1 and Table S1 provide details of each included publication.

## Identification of malnutrition in children

The recent 2023 WHO guideline on the prevention and management of wasting in infants and children under 5 years of age recommends that all infants and young children should be triaged as soon as they enter a health facility or have contact with a health worker to ensure that those with emergency or danger signs receive immediate life-saving care. In addition, the identification of nutritional status is a vital aspect of the initial assessment to ensure that children with malnutrition receive prompt and appropriate nutritional interventions (14). The WHO definitions for moderate and severe paediatric malnutrition using a single data point are well established and include weight-for-height z score, body mass index (BMI)-for-age z score, mid upper arm circumference (MUAC) z score (for all three, moderate: −2 to −2.9; severe: −3 or below), and length/height-for-age z score (moderate: −2 to −3; severe: below −3) (14). Easy-to-use tools such as the MUAC z score tape are readily available and may help with the accurate and early identification of children at risk (15, 16). In particular, the MUAC z score tape can provide a more reliable assessment in children with neurological conditions such as cerebral palsy due to the challenges with joint contractures, movement disorders and muscle atrophy. It may also be helpful in patients with fluid shifts such as ascites or oedema as well as patients with hepato-/splenomegaly or solid tumor mass where body weight may be affected (17) (see Supplementary Material).

## Malnutrition-related alterations in gastrointestinal function

The main purpose of the gastrointestinal tract is to provide nutrients to the body by digesting food into small fragments that are absorbed into the blood. Childhood malnutrition has been reported to affect digestive physiology and result in impaired reabsorption of bile salts, excessive bile salt deconjugation, pancreatic exocrine insufficiency (reduced lipase, trypsin, chymotrypsin, and amylase secretion) and impaired intestinal cell function (reduced disaccharidase content, and terminal ileal dysfunction) (6–9). In addition, intestinal bacterial overgrowth (SIBO) and diarrhoea in childhood malnutrition can result in alterations of the gut microbiota and further diarrhoea (10, 12).

**Figure 1:**
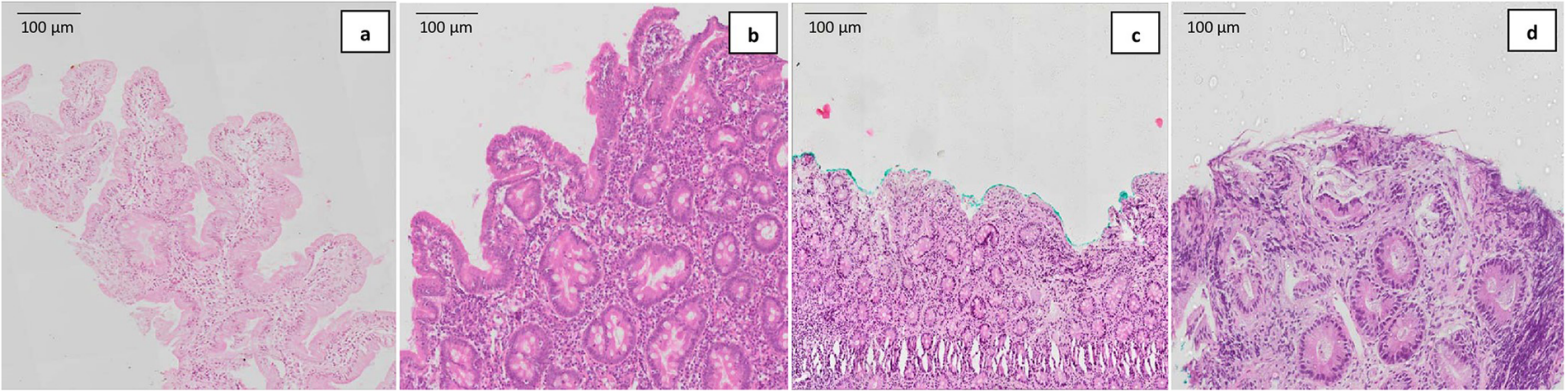
Representative histological images of: **(a)** normal villous architecture, **(b)** mild villous atrophy, **(c)** subtotal villous atrophy, and **(d)** total villous atrophy with crypt hyperplasia obtained using hematoxylin and eosin (H&E) stain in stunted Bangladeshi children aged between 12 and 18 months with possible environmental enteric dysfunction. Figure reproduced from Hossain et al. (22). This work is licensed under Creative Commons (CC by 4.0 https://creativecommons.org/licenses/by/4.0/).

The small intestine is responsible for most of the nutrient absorption and is covered in finger-like projections called villi which increase its surface area, providing a larger area for absorption to occur. Together, these microvilli form the brush border of the intestinal epithelial cells. In malnutrition, there is a histological impact which results in thinning of the intestinal wall and the mucosal lining together with reduced height of the brush border. There is also a predominance of cuboidal rather than columnar mucosal cells (18). This thinning of the intestinal wall and the mucosal lining increases gut permeability and negatively impacts the absorption of amino acids, proteins, carbohydrates, lipids, electrolytes, and other micronutrients (19, 20). Malnutrition has also been documented to be associated with altered intestinal blood flow, reduced villus height and villus atrophy, crypt hyperplasia and marked cellular infiltration (inflammation) in the lamina propria in adults (21) and in children aged between 12 and 18 months with possible environmental enteric dysfunction (22) (Figure 1). These changes result in decreased availability of absorptive surface area leading to impaired nutrient absorption.

Most of the nutrient absorption, including macronutrients, occurs in the small intestine. Carbohydrates, lipids, and proteins are absorbed differently, and malnutrition can negatively impact the absorptive capacity.

### Carbohydrate absorption

Once digested, carbohydrates are broken down into monosaccharides and absorbed into intestinal mucosal cells by either active transport (glucose and galactose) or facilitated transport (fructose). The reduced villus height and/or villus blunting observed in the small intestine of malnourished children can lead to a reduced intestinal capacity for carbohydrate digestion and absorption (23), effects that are also observed in adults (21). Furthermore, lactose intolerance may develop in some children due to secondary lactase deficiency (23, 24). The resulting accumulation of unabsorbed carbohydrates in the gastrointestinal tract leads to increased water retention within the bowel and increased flow through the bowel, causing osmotic diarrhoea (12, 23).

### Protein absorption

**Figure 2:**
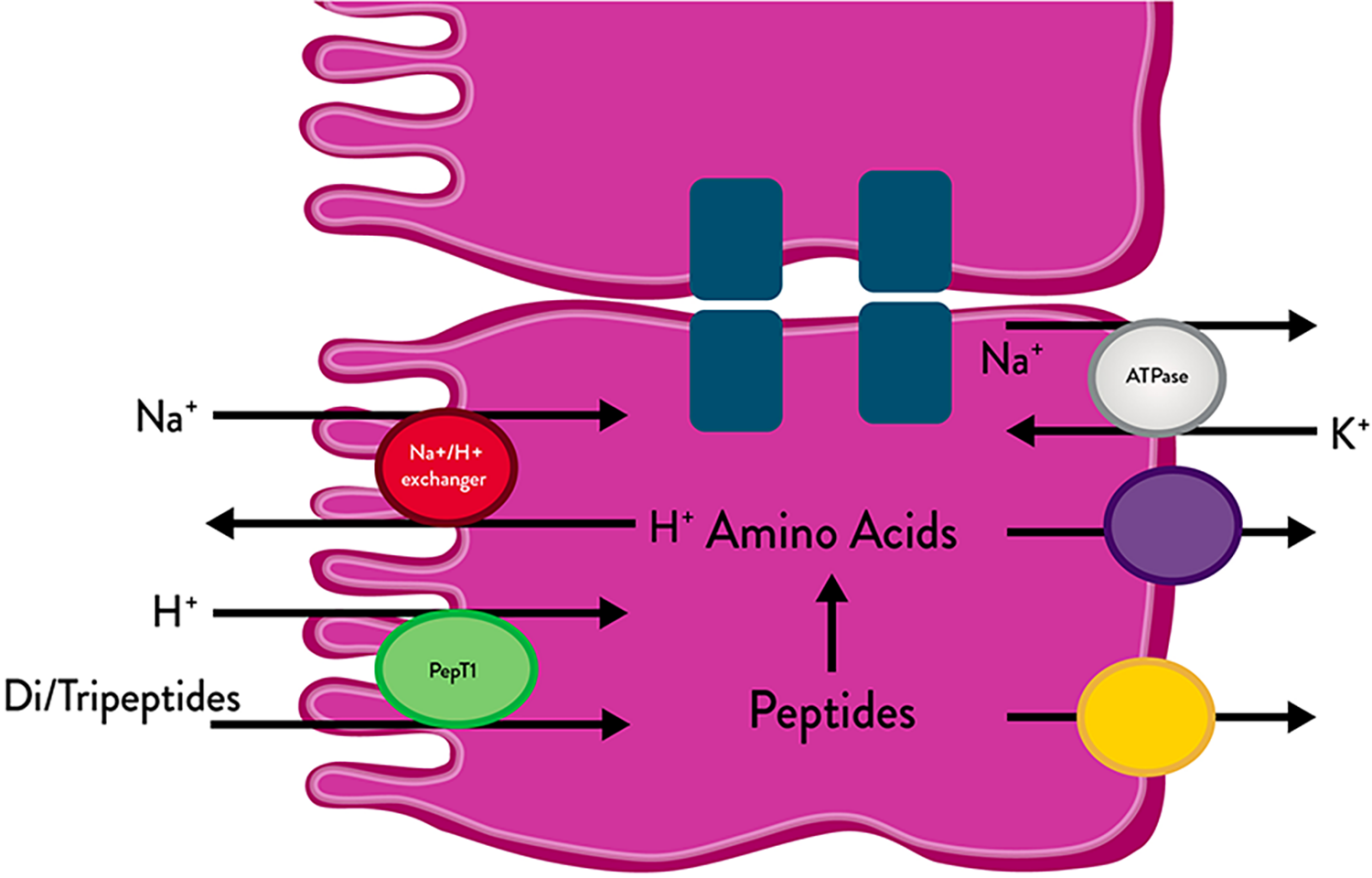
Di- and tripeptide transport across the gastrointestinal epithelium. Figure adapted from Jackman et al. (25) and Adibi (26).

Absorption of peptides and amino acids occurs in the intestine via a dual protein-carrier system (Figure 2) consisting of two separate, independent, non-competing transport systems. One system carries free amino acids that are absorbed via active transport across the intestinal wall and then enter the portal vein; the other carries di- and tripeptides that are absorbed across the intestinal wall where they are hydrolysed by intracellular peptidases into amino acids and then absorbed into the portal vein (27). The two protein carrier systems operate independently and noncompetitively, meaning the efficiency of one system does not affect the other. Hydrolyzed protein systems, which contain both free amino acids and short-chain peptides, utilise these independent carrier systems to deliver protein-derived nitrogen into the portal circulation more rapidly than formulas containing only free amino acids or intact protein. This enhances nitrogen absorption.

In malnourished children, protein maldigestion and malabsorption are considered likely due to pancreatic insufficiency (i.e., when there is a reduction in the production of pancreatic enzymes and hence reduced digestion and absorption of nutrients) and villus atrophy in the small intestine, as well as increased protein loss due to increased intestinal permeability (20). The resultant deficiency of specific essential amino acids can further worsen gastrointestinal mucosal atrophy and so reduce protein absorption even further (11, 18). Malnutrition has dramatic effects on small intestinal mucosal structure and transport function. Peptide transport is less affected by malnutrition, injury, and disease than free amino acid transport.

In pathological states with impaired mucosal absorption, peptide absorption is less severely affected than free amino acid absorption (28). One possible reason for this is because peptide transport systems are more efficient and rapid than the uptake of free amino acids (29). The existence of specific peptide carrier systems in the intestinal brush border that are independent of free amino acid carrier systems are thought to be of importance in the effective treatment of patients with intestinal malabsorption (30, 31).

In addition to the efficient and rapid uptake of di- and tripeptides compared with free amino acids, studies have indicated an improved gastrointestinal tolerance with peptide-based diets, with the rate of absorption and the degree of tolerance being dependent on the presence of small molecular weight di- and tripeptides (28).

### Fat absorption

Long chain triglycerides (LCTs) are hydrolysed within the lumen of the small intestine by the pancreatic enzyme lipase through bile salts. The resulting products, bile salts and lecithin form mixed micelles that are absorbed into the enterocytes. Fatty acids and monoglycerides are absorbed into the enterocytes where they are re-synthesized into triglycerides and packaged into chylomicrons that are transported to the blood via the thoracic duct. Medium-chain triglycerides (MCTs) are absorbed by a different process, being hydrolysed more rapidly and effectively, and since the process does not depend on pancreatic lipolysis, go directly to the liver via the portal vein.

When this fat absorption step fails, steatorrhea develops. Protein intake can affect the severity of steatorrhea and the levels of triglycerides and free fatty acids in the faeces, suggesting that a change in lipolytic activity is not the primary reason for the increase in fat in the faeces that is observed in malnutrition (18). Furthermore, abnormalities in the gastrointestinal handling of lipids together with impaired lipid solubilization and/or hydrolysis can contribute to the malabsorption of fats (32). Bacterial overgrowth in the small bowel in children can also contribute to changes in fat absorption (32). In addition, diarrhoea-related reduction in the concentrations of conjugated bile acids observed in malnutrition leads to steatorrhea (18).

Due to the impact of protein deficiency on fat absorption, improvement in fat absorption is thought to occur concomitantly with protein repletion, reaching normality in the absence of diarrhoea and after restoration of body protein (18, 33).

## Nutritional support in children with malnutrition

The WHO has clear guidance with a stepwise approach to the management of both chronic and acute malnutrition, together with that of refeeding syndrome which may occur in severely malnourished patients receiving nutritional rehabilitation (orally, enterally or parenterally) (14, 34).

Appropriate nutritional support in malnourished children is critical for long-term development and survival. Metabolic disturbances such as refeeding syndrome or hypoglycemia may occur when rapid and excessive food intake is given to severely malnourished individuals. Refeeding syndrome reflects the change from catabolic to anabolic metabolism and may cause serious clinical complications and pathophysiological changes such as hypophosphatemia, hypomagnesemia, hypokalemia, vitamin deficiency and fluid retention during refeeding (35, 36).

Monitoring key parameters such as vital functions, fluid balance, plasma biochemistry and urinary electrolytes, heart rate, ventilatory functions, and blood gases is of paramount importance during the refeeding phase of nutritional support. It is critical that energy, protein and micronutrient intakes are adjusted, depending on the phase of the rehabilitation, as per WHO guidelines (14), and gradually increased until daily nutritional requirements, including those for catch-up growth are met (35, 36). As previously mentioned, a number of different types of feed are available.

### Whole protein feeds

Most data have been published on the use of whole protein feeds in malnutrition and these are considered the first-line treatment option (14, 37). However, as a result of the impaired gastrointestinal function and severe mucosal abnormalities and malabsorption observed in malnourished children, tolerability can be an issue in some children receiving whole protein feeds leading to gastrointestinal symptoms including nausea, vomiting, alteration of bowel movements, gastroesophageal reflux symptoms, and/or abdominal pain (38). Many children with DRM also have underlying diagnoses that impair gastrointestinal function and further exacerbate the problem of tolerance. For example, children with congenital heart disease suffer from protein-losing enteropathy and abnormal gut perfusion and/or hypoxemia to the gut which can lead to dysbiosis and intestinal barrier dysfunction (39, 40). Such tolerability issues and symptoms have been reported to frequently interrupt enteral nutrition undertaken either in the home or hospital setting (38, 41–43).

Once a patient develops intolerance to a whole protein feed the best clinical approach remains undetermined, with lack of consensus guidance due to limited data in the literature. Dilution of feeds to achieve adequate tolerance and to prevent diarrhoea is not an appropriate approach in malnourished children given that they are already nutritionally compromised (44, 45). Providers may utilize strategies to reduce diarrhoea such as adding or removing fiber, slowing down feeding rate, adding pre- or probiotics, or changing to alternative formulas, which may includacid or peptide-based feeds (46, 47). At present, there is paucity of data to define which type of feed should be used second line when intolerance to a whole protein feed develops.

### Elemental or amino acid feeds

Feeds consisting of protein in the form of free amino acids are available (28, 48). These feeds are primarily used for the treatment of cow's milk protein allergy (CMPA) and other gastrointestinal conditions, and not specifically for malnutrition, where absorption of free amino acids has been shown to be less efficient than peptide absorption (13, 28). Intestinal inflammation in food allergy responds well to exclusion of the offending dietary antigen or, if the offending antigen is unknown, a hypoallergenic elemental feed composed of single amino acids (49). However, a study in 95 children aged 6–23 months with complicated severe acute malnutrition reported no difference in the level of intestinal inflammation or clinical benefits in those randomized to either a standard feed, an elemental feed or a polymeric feed for 14 days (50). Amino acid-based feeds can induce vomiting, diarrhoea and electrolyte imbalance as a result of their high osmolality (28, 51) so require careful monitoring when used.

### Peptide-based feeds

The development of peptide-based feeds is a significant milestone in the advancement of nutritional care of nutritionally compromised patients. Peptide-based feeds contain protein in the form of di- and tripeptides, which are absorbed via specific and discrete uptake systems in the gastrointestinal tract that are considered to be more rapid and efficient than those for free amino acids (52). There are emerging data to support the use of peptide-based feeds in patients who are intolerant to whole protein feeds in paediatric (and adult) populations (38, 53–55). Peptide-based feeds are associated with improved gastrointestinal tolerance, a lower risk of diarrhoea, and a better maintained and/or restored gut integrity compared with free amino acid or whole protein feeds (54, 56). Studies have suggested improved weight gain and growth associated with peptide-based feeds in different paediatric populations (54, 56, 57) (Table 1). Additionally, there may be some benefit in the management and prevention of functional gastrointestinal disorders, and it is thought that peptide-based feeds might be closer to the protein composition of human milk than intact cow's milk protein (59, 60).

**Table 1:** Summary of peptide-based feeds clinical positive results on growth and tolerance.

	Study population	Design	Feeding mode	No patients	Feeding duration	Results
Ibrahim et al., (Original paper) (54)	Critically ill children (PICU)	Single blind case control study	Standard polimeric formula (group 1) vs. peptide-based formula (group 2)	Group 1: 90 Group 2: 90	During PICU Stay (around 12 days)	Weight gain during PICU stay increased (*p* = 0.045) Duration to reach full caloric requirements decreased (*p* value = 0.001) Frequency and duration of feeding interruption decreased (*p* value = 0.001) Feeding intolerance (gastric residual volume, Abdominal distension, Vomiting, Hematemesis) decreased (*p* values <0.025) Sepsis days decreased (*p* value = 0.028)
Alexander et al., (Review paper) (56)						
Polk et al. (58)	Children with Crohńs disease	Prospective cross-over	Isotonic hydrolyzed whey formula administered via nocturnal nasogastric infusion	6 (6, served as own controls)	Intermittent diet program for 1 year	Height increased *P* < 0.0001 Weight increased *P* < 0.02
Kansu et al. (Original paper) (57)	Children with Cerebral Palsy and previous tube feeding intolerance on standard enteral formula	Prospective observational study	Enteral tube feeding via specialized peptide-based formula	96	6 months	Improvements in triceps skinfold thickness (*p* = 0.002), MUAC (*p* < 0.001) and WFH z (*p* = 0.001) scores compared with baseline. Reduction in rate and severity of intolerance symptoms apart from residue (*p* < 0.001) along with a significant decrease in type 1 (*p* < 0.001) and a significant increase in type 4 (*p* < 0.001) stool patterns

Often the increase in energy density of feeds is achieved by increasing the lipid content, but caution is needed as children with severe malnutrition have dysfunctional lipid metabolism (32). The fat component in peptide-based feeds, which can vary considerably in type and amount, is often in the form of MCTs, which have a smaller molecular weight than LCTs and can be hydrolysed faster and more completely (38).

The carbohydrate component of peptide-based feeds mainly consists of glucose oligosaccharides, which are metabolized during luminal hydrolysis and mucosal absorption (28). However, the stimulation of insulin secretion by carbohydrates in malnourished children may result in the development of refeeding syndrome. Individual circumstances including the phase of disease should be considered when initiating nutritional support to avoid overfeeding (35, 36, 61).

It should be noted that some peptide-based feeds do not contain any fiber, but different types of fiber can be added separately if required (see Supplementary Material).

## Conclusions and clinical practice considerations

Malnutrition in children is often associated with a considerable number of pathophysiological gastrointestinal changes, including increased permeability, inflammation, malabsorption and diarrhoea. These changes in gastrointestinal function observed in malnourished children negatively impact the efficacy of nutritional support and increase the likelihood of patients initially experiencing worsening gastrointestinal symptoms, both of which combine to increase the time taken to recover from malnutrition. Hence, when choosing nutritional therapy in malnourished children with compromised gastrointestinal function, consideration should be given to gut-protective interventions that promote better treatment tolerance, are appropriate for the phase of the acute stress response (62) and improve long-term outcomes.

Whilst a whole protein feed is currently the first-line treatment for malnutrition, the use of peptide-based feeds in malnourished children have been associated with improved gastrointestinal tolerance and absorption, reduced diarrhoea, reduced inflammation and improved growth and have maintained/restored gut integrity compared with free amino acids and whole-protein feeds. This is believed to be due to their rapid and efficient absorption (28, 44, 54, 56). Peptide feeds currently face limitations such as higher costs and lower availability compared to whole protein feeds. However, emerging data indicate that peptide feeds may improve clinical outcomes, reduce healthcare utilization, and potentially lower overall care costs (63). The combined characteristics of more efficient uptake of di- and tripeptides and a lower osmolality of peptide feeds compared to amino acid feeds may be advantageous for enteral nutrition management of various disease states (56).

At present, due to a lack of research in this area, gaps in clinical practice exist. As clinical practice often relies on past experience for decision making in malnourished children, the sharing of best practices between healthcare professionals from different specialties may be beneficial as the role of peptide-based feeds becomes more established. Further research in this area is needed to fill these knowledge gaps.

## Data Availability

The original contributions presented in the article are included in the article/Supplementary Material, further inquiries can be directed to the corresponding author.
