# Supplementary material for: Gastrointestinal changes in paediatric malnutrition that may impact on nutrition choice
Source: Front Pediatr. 2025 Mar 10;13:1523613. doi: 10.3389/fped.2025.1523613 (PMC11931439; doi:10.3389/fped.2025.1523613)
Supplement: Supplementary file 1 [file Supplementaryfile1.docx]

**Supplementary Material**

**Supplementary Figure 1:** Published literature flow diagram.


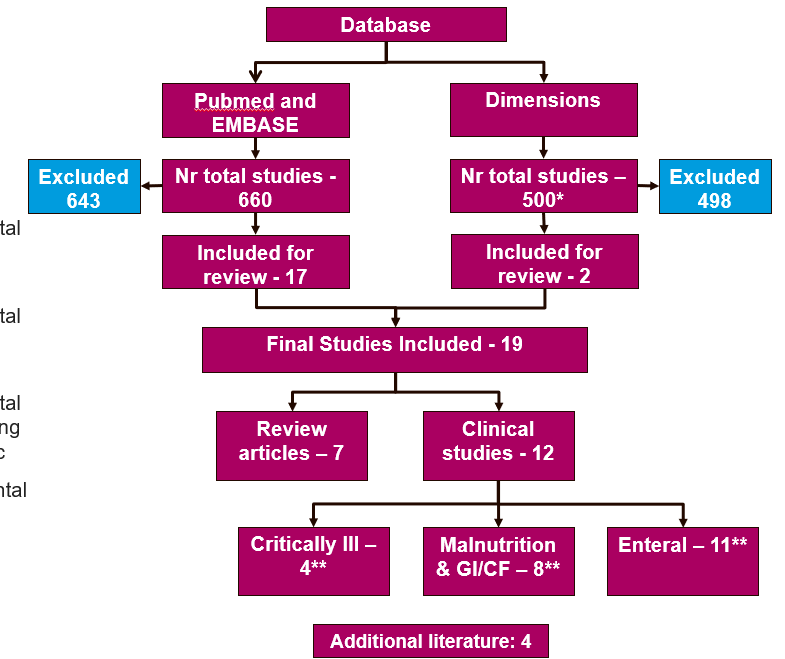


* Up to 2000 similar documents could be found, the search was limited to 500, ordered by relevance.
** Some articles classified in more than one category.

**Supplementary Table 1:** Search strategy overview

| Search strategy | No of Articles | Inclusion criteria | Exclusion criteria | No of Relevant Articles | References | Type of Study |
| --- | --- | --- | --- | --- | --- | --- |
| PubMed "Peptide-based formula" AND "children" | 16 | Population: children with malnutrition or at risk of it Peptide-based formulas Relevant outcomes | Population: adolescent or adults Not peptide-formula Irrelevant outcomes | 4 | 1.Ibrahim H, Mansour M, El Gendy YG. Peptide-based formula versus standard-based polymeric formula for critically ill children: is it superior for patients’ tolerance? Archives of Medical Science. 2020;16(3):592-6. | Single blind, case-control study |
|  |  |  |  |  | 2. Selimoglu MA, Kansu A, Aydogdu S, Sarioglu AA, Erdogan S, Dalgic B, et al. Nutritional Support in Malnourished Children With Compromised Gastrointestinal Function: Utility of Peptide-Based Enteral Therapy. Front Pediatr. 2021;9:610275. | Review |
|  |  |  |  |  | 3. Elfadil OM, Shah RN, Hurt RT, Mundi MS. Peptide‐based formula: Clinical applications and benefits. Nutrition in Clinical Practice. 2023;38(2):318-28. | Review |
|  |  |  |  |  | 4. Simpson K. Clinical Case Reports on the acceptability and tolerance of a High-Energy whey peptide-based Pediatric oral nutritional supplement in children aged over 12 months. Clin Case Rep. 2021;9(10):e04887. | Case Reports (2) |
| PubMed "Peptide-based formula" AND "children" AND "malnutrition" | 5 | Population: children with malnutrition or at risk of it Peptide-based formulas Relevant outcomes | Not peptide-based formula Irrelevant outcomes | 3 | 1.Ibrahim H, Mansour M, El Gendy YG. Peptide-based formula versus standard-based polymeric formula for critically ill children: is it superior for patients’ tolerance? Archives of Medical Science. 2020;16(3):592-6. | Single blind, case-control study |
|  |  |  |  |  | 2. Selimoglu MA, Kansu A, Aydogdu S, Sarioglu AA, Erdogan S, Dalgic B, et al. Nutritional Support in Malnourished Children With Compromised Gastrointestinal Function: Utility of Peptide-Based Enteral Therapy. Front Pediatr. 2021;9:610275. | Review |
|  |  |  |  |  | 3. Elfadil OM, Shah RN, Hurt RT, Mundi MS. Peptide‐based formula: Clinical applications and benefits. Nutrition in Clinical Practice. 2023;38(2):318-28 | Review |
| PubMed "Peptide feed" AND "children" AND "malnutrition" | 6 | / | Population: infant and adults Not peptide feed Irrelevant outcomes | 0 |  |  |

| Search strategy | No of Articles | Inclusion criteria | Exclusion criteria | No of Relevant Articles | References | Type of Study |
| --- | --- | --- | --- | --- | --- | --- |
| PubMed "Peptide formula" AND "children" AND "malnutrition" | 35 | Population: children with malnutrition or at risk of it Peptide-based formulas Relevant outcomes | Population: infant and adults and/or not malnourished children Not peptide-based formula Irrelevant outcomes | 4 | 1.Ibrahim H, Mansour M, El Gendy YG. Peptide-based formula versus standard-based polymeric formula for critically ill children: is it superior for patients’ tolerance? Archives of Medical Science. 2020;16(3):592-6. | Single blind, case-control study |
|  |  |  |  |  | 2. Selimoglu MA, Kansu A, Aydogdu S, Sarioglu AA, Erdogan S, Dalgic B, et al. Nutritional Support in Malnourished Children With Compromised Gastrointestinal Function: Utility of Peptide-Based Enteral Therapy. Front Pediatr. 2021;9:610275. | Review |
|  |  |  |  |  | 3. Elfadil OM, Shah RN, Hurt RT, Mundi MS. Peptide‐based formula: Clinical applications and benefits. Nutrition in Clinical Practice. 2023;38(2):318-28. | Review |
|  |  |  |  |  | 4. Huang XJ, Guo FF, Li F, Zhao JC, Fan YZ, Wang N, et al. [Nutritional support in children with pneumonia on mechanical ventilation by short-peptide enteral nutrition formula]. Zhongguo Dang Dai Er Ke Za Zhi. 2020;22(11):1209-14. | RCT |
| PubMed "Peptide formula" AND "pediatric" AND "malnutrition" | 27 | Population: children with malnutrition or at risk of it Peptide-based formulas Relevant outcomes | Population: infant and adults and/or not malnourished children Not peptide-based formula Irrelevant outcomes | 4 | 1.Ibrahim H, Mansour M, El Gendy YG. Peptide-based formula versus standard-based polymeric formula for critically ill children: is it superior for patients’ tolerance? Archives of Medical Science. 2020;16(3):592-6. | Single blind, case-control study |
|  |  |  |  |  | 2. Selimoglu MA, Kansu A, Aydogdu S, Sarioglu AA, Erdogan S, Dalgic B, et al. Nutritional Support in Malnourished Children With Compromised Gastrointestinal Function: Utility of Peptide-Based Enteral Therapy. Front Pediatr. 2021;9:610275. | Review |
|  |  |  |  |  | 3. Elfadil OM, Shah RN, Hurt RT, Mundi MS. Peptide‐based formula: Clinical applications and benefits. Nutrition in Clinical Practice. 2023;38(2):318-28. | Review |
|  |  |  |  |  | 4. Huang XJ, Guo FF, Li F, Zhao JC, Fan YZ, Wang N, et al. [Nutritional support in children with pneumonia on mechanical ventilation by short-peptide enteral nutrition formula]. Zhongguo Dang Dai Er Ke Za Zhi. 2020;22(11):1209-14. | RCT |

| Search strategy | No of Articles | Inclusion criteria | Exclusion criteria | No of Relevant Articles | References | Type of Study |
| --- | --- | --- | --- | --- | --- | --- |
| PubMed "Peptide feed" AND "pediatric" | 166 | / | Population: infant, neonatal or animals Not peptide feed | 0 |  |  |
| PubMed "Peptide feed" AND "pediatric" AND "malnutrition" | 3 | / | Population: not malnourished children Not peptide feed | 0 |  |  |
| PubMed "Peptide formula" AND "children or pediatric" AND "malabsorption" | 25 | Population: children with malnutrition or at risk of it Peptide-based formulas Relevant outcomes | Population: Infant, adults, animals and not malnourished children Not peptide formulas | 2 | 1. Suharyono. Short chain peptide in the treatment of chronic diarrhoea with protein maldigestion/malabsorption. Paediatr Indones. 1990;30(5-6):147-53. | Clinical trial |
|  |  |  |  |  | 2. Selimoglu MA, Kansu A, Aydogdu S, Sarioglu AA, Erdogan S, Dalgic B, et al. Nutritional Support in Malnourished Children With Compromised Gastrointestinal Function: Utility of Peptide-Based Enteral Therapy. Front Pediatr. 2021;9:610275. | Review |
| PubMed "Peptide feed" AND "children or pediatrc" AND "malabsorption" | 7 | / | Population: infant, adults and animals Not peptide feed | 0 |  |  |

| Search strategy | No of Articles | Inclusion criteria | Exclusion criteria | No of Relevant Articles | References | Type of Study |
| --- | --- | --- | --- | --- | --- | --- |
| ResearchGate (peptide formula OR peptide-based formula OR peptide feed) AND (children OR pediatric OR paediatric) AND (malnutrition OR malabsorption) | 100 | Population: children with malnutrition or at risk of it Peptide-based formulas or feed Relevant outcomes | Population: infant, adults and animals Not peptide formulas Irrelevant outcomes | 7 | 1.Ibrahim H, Mansour M, El Gendy YG. Peptide-based formula versus standard-based polymeric formula for critically ill children: is it superior for patients’ tolerance? Archives of Medical Science. 2020;16(3):592-6. | Single blind, case-control study |
|  |  |  |  |  | 2. Selimoglu MA, Kansu A, Aydogdu S, Sarioglu AA, Erdogan S, Dalgic B, et al. Nutritional Support in Malnourished Children With Compromised Gastrointestinal Function: Utility of Peptide-Based Enteral Therapy. Front Pediatr. 2021;9:610275. | Review |
|  |  |  |  |  | 3. Elfadil OM, Shah RN, Hurt RT, Mundi MS. Peptide‐based formula: Clinical applications and benefits. Nutrition in Clinical Practice. 2023;38(2):318-28. | Review |
|  |  |  |  |  | 4. Huang XJ, Guo FF, Li F, Zhao JC, Fan YZ, Wang N, et al. [Nutritional support in children with pneumonia on mechanical ventilation by short-peptide enteral nutrition formula]. Zhongguo Dang Dai Er Ke Za Zhi. 2020;22(11):1209-14. | RCT |
|  |  |  |  |  | 5. Mostafa MS, Gamal Y, Soliman MH. Peptide-based enteral formula vs a whole protein enteral formula after major intestinal surgeries in children. Annals of Pediatric Surgery. 2021;17(1):41. | Retrospective cohort study |
|  |  |  |  |  | 6. Kristyn F, Heather G. Clinical Use of Peptide-Based Formula (Peptamen Junior®, Nestle) in the Paediatric Population. International Journal of Nutrition. 2022;6(4):35-45. | Retrospective observational |
| Dialog and ProQuest. Databases Medline and Embase (peptide-based formula OR peptide formula OR peptide feed) AND (children OR pediatric OR paediatric) AND (malnutrition | 57 | Population: children with malnutrition or at risk of it Peptide-based formulas or feed Relevant outcomes | Population: infant and adults Not peptide formulas Irrelevant outcomes | 4 | 1.Ibrahim H, Mansour M, El Gendy YG. Peptide-based formula versus standard-based polymeric formula for critically ill children: is it superior for patients’ tolerance? Archives of Medical Science. 2020;16(3):592-6. | Single blind, case-control study |
|  |  |  |  |  | 2. Selimoglu MA, Kansu A, Aydogdu S, Sarioglu AA, Erdogan S, Dalgic B, et al. Nutritional Support in Malnourished Children With Compromised Gastrointestinal Function: Utility of Peptide-Based Enteral Therapy. Front Pediatr. 2021;9:610275. | Review |
|  |  |  |  |  | 3. Elfadil OM, Shah RN, Hurt RT, Mundi MS. Peptide‐based formula: Clinical applications and benefits. Nutrition in Clinical Practice. 2023;38(2):318-28. | Review |
|  |  |  |  |  | 4. Huang XJ, Guo FF, Li F, Zhao JC, Fan YZ, Wang N, et al. [Nutritional support in children with pneumonia on mechanical. | RCT |

| Search strategy | No of Articles | Inclusion criteria | Exclusion criteria | No of Relevant Articles | References | Type of Study |
| --- | --- | --- | --- | --- | --- | --- |
| OR malabsorption) |  |  |  |  | ventilation by short-peptide enteral nutrition formula]. Zhongguo Dang Dai Er Ke Za Zhi. 2020;22(11):1209-14 |  |
| Dialog and ProQuest. Databases Medline and Embase (semi-elemental formula) OR (semi-elemental feed) AND (malnutrition OR undernutrition OR failure to thrive OR faltering growth OR absorption OR tolerance ) AND (children OR pediatric OR paediatric) | 127 | Population: children Semi-elemental formulas or feed Relevant outcomes | Population: infant, adults and animals Not semi-elemental formulas or feed Irrelevant outcomes | 7 | 1. Odinaeva ND, Kondratyeva EI, Maksimycheva TY. The first experience of using a ready-to-use liquid form of a hypercaloric semi-elemental product for enteral nutrition in children with cystic fibrosis. Voprosy praktičeskoj pediatrii. 2023;18(1):55-61. | Retrospective Observational(Single-center ) |
|  |  |  |  |  | 2. Verduci E, Salvatore S, Bresesti I, Di Profio E, Pendezza E, Bosetti A, et al. Semi-Elemental and Elemental Formulas for Enteral Nutrition in Infants and Children with Medical Complexity-Thinking about Cow's Milk Allergy and Beyond. Nutrients. 2021;13(12). | Review |
|  |  |  |  |  | 3. Vandenplas Y, Plaskie K, Hauser B. Safety and adequacy of a semi-elemental formula for children with gastro-intestinal disease. Amino Acids. 2010;38(3):909-14 | Prospective, open trial |
|  |  |  |  |  | 4. Eichenberger JR, Hadorn B, Schmidt BJ. A semi-elemental diet with low osmolarity and high content of hydrolyzed lactalbumin in the treatment of acute diarrhea in malnourished children. Arq Gastroenterol. 1984;21(3):130-5. | RCT |
|  |  |  |  |  | 5. Sutphen JaA-SA. Nutritional Management of Pediatric Short Bowel Syndrome. Practical gastroenterology. 2003. | Review |
|  |  |  |  |  | 6. Mohamed Elfadil O, Steien DB, Narasimhan R, Velapati SR, Epp L, Patel I, et al. Transition to peptide-based diet improved enteral nutrition tolerance and decreased healthcare utilization in pediatric home enteral nutrition. Journal of Parenteral and Enteral Nutrition. 2022;46(3):626-34. | Review |
|  |  |  |  |  | 7. Alexander DD, Bylsma LC, Elkayam L, Nguyen DL. Nutritional and health benefits of semi-elemental diets: A comprehensive summary of the literature. World J Gastrointest Pharmacol Ther. 2016;7(2):306-19. | Review |

| Search strategy | No of Articles | Inclusion criteria | Exclusion criteria | No of Relevant Articles | References | Type of Study |
| --- | --- | --- | --- | --- | --- | --- |
| Dialog and ProQuest. Databases Medline and Embase (hydrolysed formula) OR (hydrolysed feed) AND (malnutrition OR undernutrition OR failure to thrive OR faltering growth OR absorption OR tolerance ) AND (children OR pediatric OR paediatric) Subjects: [Clear(Clear Subject) Subject]: NOT (infant AND infant formula AND infant, newborn AND newborn AND infant food AND adult AND infant feeding AND infant nutrition AND adolescent AND infancy AND infant nutritional physiological phenomena AND infant, premature AND middle aged AND infantile colic AND infant disease AND young adult MeSH Subjects: [Clear(Clear MeSH subjects) MeSH subjects]: Humans OR Child OR Child, Preschool Document type: [Clear(Clear Document type) Document type]: Journal Article OR Review OR Randomized Controlled Trial OR Systematic Review OR Clinical Trial OR Meta-Analysis OR Observational Study OR Evaluation Study | 86 | Population: children with allergies Hydrolysed formulas or feed Relevant outcomes | Population: not children Not hydrolysed formulas or feed Majority of them partially or extensively hydrolysed formulas Irrelevant outcomes | 2 The "relevant" articles are related to allergies | 1. Strozyk A, Horvath A, Meyer R, Szajewska H. Efficacy and safety of hydrolyzed formulas for cow's milk allergy management: A systematic review of randomized controlled trials. Clin Exp Allergy. 2020;50(7):766-79. | Systematic review of RCTs |
|  |  |  |  |  | 2. Nocerino R, Di Costanzo M, Bedogni G, Cosenza L, Maddalena Y, Di Scala C, et al. Dietary Treatment with Extensively Hydrolyzed Casein Formula Containing the Probiotic Lactobacillus rhamnosus GG Prevents the Occurrence of Functional Gastrointestinal Disorders in Children with Cow's Milk Allergy. J Pediatr. 2019;213:137-42.e2. | Cohort |

| Search strategy | No of Articles | Inclusion criteria | Exclusion criteria | No of Relevant Articles | References | Type of Study |
| --- | --- | --- | --- | --- | --- | --- |
| Additional search: Dimensions.  (by Abstract - *Odinaeva et al.* 2023) | 500 | Population: children with malnutrition or at risk of it Semi-elemental formulas or feed Relevant outcomes | Population: not malnourished children, infant or adults Not semi-elemental formulas or feed Irrelevant outcomes | 2 | 1. Maksimycheva TY, Kondratyeva EI, Odinaeva ND. Experience of using semi-elemental formulas for enteral nutrition in children with cystic fibrosis. Meditsinskiy sovet = Medical Council. 2021(1):228-34. | Prospective Observational, without comparison group |
|  |  |  |  |  | 2. Bushueva TV, Borovik T, Roslavtseva E, Shen N, Simonova O, Burkina N, et al. The effectiveness of the use of a specialized formula based on hydrolyzed milk protein in children older than 1 year of age with cystic fibrosis. Russian Pediatric Journal. 2022;25:12-7. | Clinical trial |
| Additional references |  |  |  | 3 | 1. Smith C, McCabe H, Macdonald S, Morrison L, Prigg R, Trace S, et al. Improved growth, tolerance and intake with an extensively hydrolysed peptide feed in infants with complex disease. Clin Nutr. 2018;37(3):1005-12. | Prospective, interventional |
|  |  |  |  |  | 2. Marino LV, Eveleens RD, Morton K, Verbruggen S, Joosten KFM. Peptide nutrient-energy dense enteral feeding in critically ill infants: an observational study. J Hum Nutr Diet. 2019;32(3):400-8. | Retrospective, observational |
|  |  |  |  |  | 3. A. Kansu and TolerUPStudy Group. Utility of a specialized peptide-based enteral formula containing medium-chain triglycerides in provision of enteral tube feeding in children with cerebral palsy: data from prospective observational tolerup study | Prospective, observational |

**Supplementary Table 2:** Relevant articles identified from literature search

| Title | Author(s) | Year | Aims/objectives |
| --- | --- | --- | --- |
| Nutritional Support in Malnourished Children With Compromised Gastrointestinal Function: Utility of Peptide-Based Enteral Therapy | M. A. Selimoglu, A. Kansu, S. Aydogdu, A. A. Sarioglu, S. Erdogan, B. Dalgic, A. Yuce and F. Cullu Cokugras | 2021 | This review focuses on nutritional support in malnourished children with compromised gastrointestinal function addressing the interplay between malnutrition and gastrointestinal dysfunction, and the specific role of peptide-based enteral therapy in pediatric malnutrition. |
| Peptide‐based formula: Clinical applications and benefits | O. M. Elfadil, R. N. Shah, R. T. Hurt and M. S. Mundi | 2023 | This review aims to navigate through key clinical applications and benefits of peptide-based formulas and to discuss relevant data shared in the literature. |
| Peptide-based formula versus standard-based polymeric formula for critically ill children: is it superior for patients’ tolerance? | H. Ibrahim, M. Mansour and Y. G. El Gendy | 2020 | The aim of this study was to compare the effect of a peptide-based formula versus a standard polymeric formula on feeding tolerance and whether this will affect the outcome among critically ill children. |
| Transition to peptide-based diet improved enteral nutrition tolerance and decreased healthcare utilization in pediatric home enteral nutrition | O. Mohamed Elfadil, D. B. Steien, R. Narasimhan, S. R. Velapati, L. Epp, I. Patel, J. Patel, R. T. Hurt and M. S. Mundi | 2022 | The objective was to assess gastrointestinal tolerance and impact on healthcare utilization in children receiving peptide-based diet.. |
| Peptide-based enteral formula vs a whole protein enteral formula after major intestinal surgeries in children | M. S. Mostafa, Y. Gamal and M. H. Soliman | 2021 | The aim was to compare the results and outcome regarding tolerance, nutritional status, and hospital stay following a postoperative diet of peptide-based enteral formula against a whole protein enteral formula after major intestinal surgeries in pediatric patients who had resection and re-anastomosis after intussusception. |
| Clinical Case Reports on the acceptability and tolerance of a High-Energy whey peptide-based Pediatric oral nutritional supplement in children aged over 12 months | K. Simpson | 2021 | The nutritional management of the complex needs of children with impaired gastrointestinal function can be challenging, using a high-energy pediatric whey-based peptide formula in clinical practice demonstrates its role in managing symptoms. |
| Title | **Author(s)** | **Year** | **Aims/objectives** |
| [Nutritional support in children with pneumonia on mechanical ventilation by short-peptide enteral nutrition formula] | X. J. Huang, F. F. Guo, F. Li, J. C. Zhao, Y. Z. Fan, N. Wang and J. Y. Qiao | 2020 | The objective was to observe the incidence of malnutrition and nutritional risk in children with pneumonia on mechanical ventilation in the pediatric intensive care unit (PICU), and to explore the nutritional support effect of short-peptide enteral nutrition formula. |
| Clinical Use of Peptide-Based Formula (Peptamen Junior®, Nestle) in the Paediatric Population | F. Kristyn and G. Heather | 2022 | This retrospective observational audit aimed to describe the characteristics of the paediatric patients who have been prescribed a hydrolysed whey protein, medium chain triglycerides (MCT) based formula, and the nutritional outcomes. |
| Nutritional and health benefits of semi-elemental diets: A comprehensive summary of the literature | D. D. Alexander, L. C. Bylsma, L. Elkayam and D. L. Nguyen | 2016 | The aim was to critically review and summarize the literature on nutritional and health outcomes of semi-elemental formulations on various nutritionally vulnerable patient populations who are unable to achieve adequate nutrition from standard oral diets. |
| Short chain peptide in the treatment of chronic diarrhoea with protein maldigestion /malabsorption | Suharyono | 1990 | The aim was to compare a formula using short chain peptides (SCP) vs a non-SCP formula in children suffering from chronic diarrhoea with protein maldigestion/malabsorption |
| The first experience of using a ready-to-use liquid form of a hypercaloric semi-elemental product for enteral nutrition in children with cystic fibrosis | N. D. Odinaeva, E. I. Kondratyeva and T. Y. Maksimycheva | 2023 | The objective was to evaluate the dynamics of anthropometric indicators and body composition using bioimpedance analysis in children with cystic fibrosis against the background of dietary correction using a semi-elemental hypercaloric product. |
| Semi-Elemental and Elemental Formulas for Enteral Nutrition in Infants and Children with Medical Complexity-Thinking about Cow's Milk Allergy and Beyond | E. Verduci, S. Salvatore, I. Bresesti, E. Di Profio, E. Pendezza, A. Bosetti, M. Agosti, G. V. Zuccotti and E. D'Auria | 2021 | In the present narrative review, we specifically focus on the use of elemental and semi-elemental enteral formulas in preterm neonates and in patients with neurological impairments as clinical “models” of medical complexity. |

| Title | Author(s) | Year | Aims/objectives |
| --- | --- | --- | --- |
| Safety and adequacy of a semi-elemental formula for children with gastro-intestinal disease | Y. Vandenplas, K. Plaskie and B. Hauser | 2010 | This study evaluated the nutritional adequacy of a semi-elemental diet in children with functional gastro-intestinal disorders. |
| A semi-elemental diet with low osmolarity and high content of hydrolyzed lactalbumin in the treatment of acute diarrhea in malnourished children | J. R. Eichenberger, B. Hadorn and B. J. Schmidt | 1984 | The aim of this study was to evaluate a semi-elemental diet (SED) vs available proprietary formulas or diluted cow's milk with added carbohydrates. |
| Nutritional Management of Pediatric Short Bowel Syndrome | J. a. A.-S. A. Sutphen | 2003 | The aim of this paper was to assess the nutritional management of children with short bowel syndrome |
| Experience of using semi-elemental formulas for enteral nutrition in children with cystic fibrosis | T. Y. Maksimycheva, E. I. Kondratyeva and N. D. Odinaeva | 2021 | The aim was to evaluate the effectiveness of correcting the nutritional status in children with cystic fibrosis against the background of concomitant conditions using a semi-element product for enteral nutrition. |
| The effectiveness of the use of a specialized formula based on hydrolyzed milk protein in children older than 1 year of age with cystic fibrosis | T. V. Bushueva, T. Borovik, E. Roslavtseva, N. Shen, O. Simonova, N. Burkina, N. Lyabina and I. Sokolov | 2022 | The purpose was evaluation of the tolerability and effectiveness of the domestic specialized semi-elemental formula based on hydrolyzed milk protein «NUTRIEN® Elemental» in the treatment of protein-energy malnutrition in CF children older than 1 year of age with malabsorption syndrome.. |
| Efficacy and safety of hydrolyzed formulas for cow's milk allergy management: A systematic review of randomized controlled trials | A. Strozyk, A. Horvath, R. Meyer and H. Szajewska | 2020 | The objective was to summarize evidence on the efficacy and safety of the use of extensively hydrolyzed formulas (EHFs) for the treatment of children with cow's milk allergy (CMA). DESIGN: Systematic review of randomized controlled trials (RCTs) per PRISMA guidelines. The risk of bias of included RCTs was assessed using the Cochrane Collaboration's risk of bias tool. In general, a narrative synthesis of the findings was performed. When sufficient data were available, a meta-analysis using the random-effect model was performed |

| Title | Author(s) | Year | Aims/objectives |
| --- | --- | --- | --- |
| Dietary Treatment with Extensively Hydrolyzed Casein Formula Containing the Probiotic Lactobacillus rhamnosus GG Prevents the Occurrence of Functional Gastrointestinal Disorders in Children with Cow's Milk Allergy | R. Nocerino, M. Di Costanzo, G. Bedogni, L. Cosenza, Y. Maddalena, C. Di Scala, G. Della Gatta, L. Carucci, L. Voto, S. Coppola, A. M. Iannicelli and R. Berni Canani | 2019 | The objective was to investigate whether the addition of the probiotic Lactobacillus rhamnosus GG (LGG) to the extensively hydrolyzed casein formula (EHCF) for cow's milk allergy (CMA) treatment could reduce the occurrence of functional gastrointestinal disorders (FGIDs). |
| Improved growth, tolerance and intake with an extensively hydrolysed peptide feed in infants with complex disease | C. Smith, H. McCabe, S. Macdonald, L. Morrison, R. Prigg, S. Trace, J. Livingstone, J. Callan, J. Cotton, G. Hubbard and R. J. Stratton | 2018 | This study aimed to investigate the effectiveness, tolerance and acceptability of nutritional support with a specially formulated, paediatric peptide feed in infants with complex disease and signs of growth faltering with their current nutritional management.. |
| Peptide nutrient-energy dense enteral feeding in critically ill infants: an observational study | L. V. Marino, R. D. Eveleens, K. Morton, S. Verbruggen and K. F. M. Joosten | 2019 | The aim of this observational study was to characterise the use of a PEF amongst critically ill infants in two paediatric intensive care units (PICUs). |
| Utility of a specialized peptide-based enteral formula containing medium chain triglycerides in provision of enteral tube feeding in children with cerebral palsy: Data from prospective observational TolerUP Study | A. Kansu, TolerUPStudy Group | 2021 | The study aimed to evaluate the utility of enteral tube feeding (ETF) with specialized peptide-based formula (SPBEF) containing medium chain triglycerides (MTCs) in children with cerebral palsy (CP).. |

**Supplementary Table 3.** World Health Organization (WHO) classification of nutritional status of infants and children (birth to 5 years). Based on: Guideline: assessing and managing children at primary health-care facilities to prevent overweight and obesity in the context of the double burden of malnutrition. Updates for the Integrated Management of Childhood Illness (IMCI). Geneva: World Health Organization; 2017. Licence: CC BY-NC-SA 3.0 IGO.

| **Nutritional status** | **Indicator and cut off value {vs median of WHO Growth Standards (2006)}** |
| --- | --- |
| **Moderately underweight** | Weight-for-age z-score <−2 and ≥−3 |
| **Severely underweight** | Weight-for-age z-score <−3 |
| **Moderate acute malnutrition** | Weight-for-length/height z-score between −2 and −3  BMI-for-age z-score between −2 and −3  Mid-upper arm circumference (MUAC) ≥115 mm to <125 mm |
| **Severe acute malnutrition** | Weight-for-length/height z-score <−3  BMI-for-age z-score <−3  MUAC <115 mm |
| **Moderate chronic malnutrition (moderately stunted)** | Length/height-for-age z-score ≤−2 and ≥−3 |
| **Severe chronic malnutrition (severely stunted)** | Length/height-for-age z-score <−3 |
| **Moderately wasted** | Weight-for-length/height z-score ≤−2 and ≥−3 |
| **Severely wasted** | Weight-for-length/height z-score <−3 |
